# North Carolina Pharmacists’ Support for Hormonal Contraception Prescription Status Change

**DOI:** 10.1177/08971900221074966

**Published:** 2022-03-24

**Authors:** Rachel A. Parry, Gwen Seamon, Mollie Ashe Scott, Casey R. Tak

**Affiliations:** 1 Division of Pharmaceutical Outcomes and Policy, Eshelman School of Pharmacy, 2331 The University of North Carolina at Chapel Hill, Chapel Hill, NC, USA; 2 Mountain Community Health Partnership, Burnsville, NC, USA; 3 UNC Health Sciences, Mountain Area Health Education Center, Asheville, NC, USA; 4 Division of Practice Advancement and Clinical Education, Eshelman School of Pharmacy, 2331 The University of North Carolina at Chapel Hill, Chapel Hill, NC, USA; 5 Department of Pharmacotherapy, College of Pharmacy, University of Utah, Salt Lake City, UT, USA

**Keywords:** contraception, pharmacists

## Abstract

Pharmacist-prescribed hormonal contraception (HC) may offer additional avenues of access for patients; however, it is unknown whether pharmacists would support over-the-counter access to contraception over pharmacist-prescribed models. **Objective:** The objective of this study was to understand how North Carolina (NC) pharmacists believed HC should be classified and how pharmacist and pharmacy characteristics were associated with those beliefs. **Methods:** This study was a secondary analysis of a cross-sectional, anonymous, online survey completed by 587 licensed NC pharmacists. The primary outcome of interest was how pharmacists believed HC should be classified: prescription-only, pharmacist-prescribed, behind-the-counter, or over-the-counter. Multinomial bivariate and multivariable regression analyses were conducted to describe the association between pharmacist and pharmacy characteristics with the outcomes of interest through odds ratios and adjusted odds ratios, respectively. Chi-square tests were used to examine the association of geographic location with distribution of attitudes toward HC classification. **Results:** Fifty-one percent of NC pharmacists supported classification of HC as pharmacist-prescribed, while 23% supported non-prescription (behind- or over-the-counter) classification. Controlling for pharmacist demographics and pharmacy characteristics, completing residency training was significantly associated with supporting pharmacist-prescribed vs prescription-only classification (adjusted odds ratio (aOR) = 2.55, P = .02). Pharmacists had higher odds of supporting pharmacist-prescribed vs prescription-only HC if they agreed that they were well trained to do so (aOR = 3.14, P < .01). Distribution of attitudes about classification of HC did not significantly differ by geographic location (P = .14). **Conclusions:** Most NC pharmacists support deviating from the current prescription-only classification of HC, with more support for pharmacist-prescribed classification. Continuing education programs should focus on training pharmacists to feel more confident prescribing HC.

## Introduction

In 2011, there were 45 unintended pregnancies per 1000 women of reproductive age (15-44 y old), representing almost half of all pregnancies in the United States (US) that year.^1^ In 2008, women at risk of unintended pregnancy who were using no form of contraception comprised 50% of unintended pregnancies, though they represented only 10% of the women at risk of an unintended pregnancy.^2^ Significant barriers to contraception access include unaffordability, lack of refills, and being unable to reach an appointment with a clinician; these barriers prevent women who wish to avoid pregnancy from effectively or consistently utilizing hormonal contraception.^3-5^ These barriers are often more pronounced in rural areas.^6^

As one of the most accessible healthcare professionals, pharmacists are uniquely poised to increase access to self-administered hormonal contraception (HC), including oral hormonal contraception, transdermal patch, and the intravaginal ring (*National Association of Chain Drug Stores. Re: Health Care Workshop, Project No. P131207*, 2014). Currently, national discussions about increasing HC access focus on doing so through one of 2 approaches: (1) increasing or allowing pharmacist-prescribed HC and (2) moving self-administered HC over-the-counter (OTC).

In the US, where the US Food and Drug Administration (FDA) classifies HC as prescription-only, pharmacists do not have prescribing authority under their pharmacy license; “pharmacist-prescribed” HC describes a pharmacist prescribing medication(s) using a protocol authorized by the state (e.g., state-wide protocol) or with an individual physician (e.g., collaborative practice agreement). As of January 2021, eighteen states or jurisdictions have passed laws that allow pharmacists to, in varying degrees, prescribe HC through a protocol or collaborative practice agreement.^7^ These augmentations to pharmacist practice have positive outcomes for patients and payers, as prior work has found pharmacist-prescription of HC to be a cost-effective approach to avert unintended pregnancies and may promote access in high-need areas, such as rural regions.^8,9^

Alternatively, classifying contraception as OTC may increase utilization of HC by women at risk of unintended pregnancy. Eighty-one percent of women who sought abortion services at 6 urban clinics in the US supported OTC access to oral contraceptives, and, notably, 33% of women who planned on using no contraception reported that they would use oral contraceptives if they were available OTC.^10^ Globally, a study published in 2012 found that 35 countries legally allow access to HC OTC, and 11 countries allowed OTC access as long as the woman is screened by a health professional to ensure she is a good candidate.^11^ Medical associations, such as the American College of Obstetricians and Gynecologists (ACOG) and American Medical Association (AMA), agree that most contraindications can be self-screened by the women seeking oral contraceptives (*American Medical Association Resolution D-75.995 (Sub. Res. 507, A-13): Over-the-Counter Access to Oral Contraceptives*)*.*^12,13^

Contraception policy and regulation has been ever-changing in the US over the last century. The FDA approved the emergency hormonal contraception pill levonorgestrel to be available OTC to all women of childbearing age in 2013.^14^ US stakeholders have varying opinions on if or how HC classification should shift next. The AMA and ACOG support OTC access to oral contraceptives, but ACOG notes that pharmacist-prescribed classification of contraceptives may be a necessary step on the road to move HC from prescription-only to OTC (*American Medical Association Resolution D-75.995 (Sub. Res. 507, A-13): Over-the-Counter Access to Oral Contraceptives*)*.*^12,13^ A survey of medical providers, including physicians, physician assistants, and advanced practice registered nurses, in 2017 found that 74% supported expanding access to self-administered hormonal contraceptives (pill, patch, and ring) through pharmacist-initiated access; only 47%, however, supported over-the-counter access to these contraceptives.^15^ Enacting pharmacist-prescribed legislation is widely supported by pharmacists and the American Pharmacists Association (APhA), but a recent survey of pharmacists, located in a variety of states, found more than 75% of pharmacists oppose OTC hormonal contraception.^16-19^

Due to the ever-changing landscape of contraception policy in the US and the role that pharmacy plays in contraception access, research is needed to understand pharmacists’ attitudes in providing self-administered HC under its current classification, through pharmacist-prescribed mechanisms, or OTC. The objective of this study was to examine factors associated with North Carolina pharmacists’ attitudes toward changing the categorization of oral contraception to either pharmacist-prescribed or OTC and the potential impact of those attitudes on contraception access in rural areas compared to urban areas.

## Materials and Methods

### Study Design and Setting

This study was a secondary analysis of a cross-sectional, web-based survey of a convenience sample of licensed pharmacists in North Carolina.^20^ The survey was designed based on previous research and pilot-tested with a convenience sample of pharmacists before distribution. In November 2018, a recruitment email introducing and providing a link to the survey was emailed to pharmacists with an active license with the North Carolina State Board of Pharmacy (NCBOP) who were currently living in North Carolina. Data were collected using Qualtrics® software (Qualtrics, Provo, UT). Consent was obtained at the beginning of the survey. This study was determined to be exempt by the University of North Carolina at Chapel Hill Institutional Review Board.

### Measures

#### Pharmacist and Pharmacy Characteristics

The survey was designed to collect information about pharmacist attitudes toward pharmacist-prescribed hormonal contraception and barriers to implementation of a pharmacist-prescribed hormonal contraception service. Pharmacist demographics were collected categorically and dichotomously and included gender (male or female), age group (younger than 40, 40-59, or 60 and older years), years licensed as a pharmacist (< 11, 11 to 20, or > 20 y), and state from which they graduated with their pharmacy degree (North Carolina or other). Pharmacists were asked to select all completed levels of pharmacy education, which were any combination of the following: Bachelors of Science in Pharmacy (B.S.Pharm.), Doctor of Pharmacy (Pharm.D.), pharmacy residency (residency), board certifications (e.g., Board Certified Pharmacotherapy Specialist), other higher education (fellowship, Masters of Science, and PhD), and a free text box for “Other” education.

Pharmacists were asked to select the type of pharmacy in which they primarily practiced (e.g., community practice, hospital pharmacist, academia, and ambulatory care clinic). Characteristics of their primary pharmacy location were collected, including clinical services offered (e.g., vaccinations, medication therapy management, and/or emergency contraception).

Attitudes about pharmacist-prescribed HC were collected by asking participants’ level of agreement about 5 statements on a Likert scale from 1 to 5, where 1 = strongly disagree, 5 = strongly agree, and the midpoint 3 = undecided. Level of agreement with questions about pharmacists’ atttitudes that were answered on the Likert scale were dichotomized to agree (strongly agree and agree) and neutral/disagree (neutral, disagree, and strongly disagree). Statements included “Pharmacists are well-trained/educated to prescribe hormonal contraception,” “Prescribing hormonal contraception allows pharmacists to practice at a higher level,” “Increased access to hormonal contraception is an important public health issue,” “Prescribing hormonal contraception will strengthen relationships with local physicians and clinics,” and “Rural areas would benefit from pharmacist-prescribed hormonal contraception.”

#### Outcomes

The primary outcome of interest was how pharmacists believed oral contraception should be classified. Participants selected the status they believed oral contraception should be categorized: (1) prescription-only, (2) pharmacist-prescribed, (3) behind-the-counter without prescription, and (4) over-the-counter with or without age restriction. Respondents were allowed only one selection to reflect their preference. The primary outcome was divided into 3 classifications: prescription-only, pharmacist-prescribed, or non-prescription (behind-the-counter or OTC). All available outcome responses from the survey were used in the analysis.

Definitions for classifications were not provided for survey participants, but they can be understood in light of NC state laws outlining scope of pharmacist practice. For this study, “prescription-only” refers to contraception prescribed by a prescriber (eg, physician, physician assistant, nurse practitioner, and certified nurse midwife), which does not include a pharmacist in NC.^21,22^*Nurse Practitioner*^23,24^*:* “Pharmacist-prescribed” refers to pharmacists prescribing hormonal contraception under the supervision of a physician, either through a state-wide protocol or collaborative practice agreement. Currently, in NC, pharmacists can only prescribe medications through collaborative practice agreements if they receive additional training and become a registered Certified Pharmacist Practitioner (CPP) with the NCBOP, but CPPs in NC do not regularly prescribe contraception.^22^ “Behind-the-counter without a prescription” would require that patients interact with the pharmacist in order to obtain contraception, but would not require a prescription. OTC classification would not require pharmacist consultation or approval in order to obtain HC.

Geographic location was self-reported in the survey by asking the pharmacist whether the geographic location of their primary pharmacy practice site was urban, suburban, or rural.

### Data Analysis

Descriptive statistics (e.g., count and percentage) were calculated for all variables. Multinomial bivariate and multivariable regressions were conducted to identify whether pharmacists’ demographics, pharmacy characteristics, and attitudes about pharmacist-prescribed hormonal contraception were associated with the primary outcome, pharmacists’ attitudes about hormonal contraception classification. A generalized logit model was fitted with prescription-only as the referent group. To simplify the interpretation of the model, the outcome variable was condensed into 3 categories: prescription-only, pharmacist-prescribed, or non-prescription (behind-the-counter and OTC). Predictor variables included pharmacist and practice characteristics.

We first explored the individual relationship between each predictor variable and the outcome variable. Pearson correlation coefficients as well the variance inflation factor and tolerance were used to identify multicollinearity among predictors; for a pair of variables with a Pearson correlation coefficient ≥ |.8|, one of the variables was removed. Once candidate multicollinear variables were identified, we assessed their relative clinical importance to the model and removed those deemed less important. We also explored separation of data and determined which variables were nearly perfectly aligned with the outcome variable and removed those to improve model fit. Goodness-of-fit was evaluated using Hosmer and Lemeshow statistics and variance inflation factors. All analyses were conducted using SAS v9.4® (SAS Institute, Cary, NC).

## Results

The survey link was successfully delivered via email to 12,001 actively licensed pharmacists residing in North Carolina through the North Carolina Board of Pharmacy listserv. Of those, 754 pharmacists opened the link, representing a response rate of 6.3%. Pharmacist responses were excluded if they did not consent “yes” to participating in the study via a question at the beginning of the survey (n = 10) or if they did not answer how they believe oral contraception should be classified, the primary outcome of interest (n = 157). The final cohort consisted of 587 pharmacists who were mostly female (66%) and younger than 40 y old (54%, Table 1). A majority of pharmacists had a doctorate of pharmacy education level (72%) and 48% had been licensed for 10 or fewer years (see Table 1 for detailed characteristics of the study sample).

**Table 1.** Pharmacist Demographics and Pharmacy Characteristics.

	N = 587
Pharmacist Demographics	% (n)
Age	
< 40 y	53.8 (316)
4059 y	30.5 (179)
> 59 y	14.1 (83)
Missing	.3 (2)
Years as licensed pharmacist	
< 11 y	48.2 (283)
1120 y	17.4 (102)
> 20 y	34.4 (202)
Gender	
Female	65.8 (386)
Missing	.5 (3)
Type of pharmacy education (select all)	
BSPharm	31.7 (186)
PharmD	72.2 (424)
Residency	22.0 (129)
Fellowship, MS, or PhD	5.3 (31)
Board certification (categorical)	12.4 (73)
Graduated from school of pharmacy in North Carolina	62.0 (364)
Missing	1.2 (7)
Primary practice is in a community pharmacy	38.3 (225)
Pharmacy characteristics	
Clinical services offered	
Vaccination(s)	42.9 (252)
Medication therapy management	29.1 (171)
Level of privacy in counseling area	
Private	12.4 (73)
Semi-private	22.5 (132)
No private	16.4 (96)
Missing	48.7 (286)
Emergency contraception for sale	
Yes	41.6 (244)
No	8.7 (51)
Unsure	1.0 (6)
Missing	48.7 (286)

Abbreviations: BSPharm: Bachelors of Science in Pharmacy; MS: Masters of Science; PharmD: Doctor of Pharmacy; PGY: post-graduate year; PhD: Doctor of Philosophy

### Pharmacist Attitudes about Classification of Oral Contraception and Access

Approximately half of pharmacists believed that oral contraception should be classified as pharmacist-prescribed (51%), while a very small proportion believed that it should be moved to OTC or behind-the-counter (8% and 15.7%, respectively, Table 2). Distribution of pharmacist attitudes did not significantly differ by self-reported geographic location (P = .14, Table 2).

**Table 2.** Pharmacist Attitudes about Preferred Contraception Classification by Geographic Location.

	Prescription-Only	Pharmacist-Prescribed	Behind-The-Counter	Over-the-counter
Total (N = 587) % (n)	25.0 (147)	51.3 (301)	15.7 (92)	8.0 (47)
Urban (N = 174) % (n)	23.6 (41)	56.9 (99)	12.1 (21)	7.5 (13)
Suburban (N = 227) % (n)	23.8 (54)	50.2 (114)	17.2 (39)	8.8 (20)
Rural (N = 163) % (n)	28.2 (46)	47.9 (78)	18.4 (30)	5.5 (9)
p-value	.1395			

N = 23 missing or prefer not to answer

### Predictors of Attitudes about Classification of Oral Contraception

In the final multivariate multinomial regression model, pharmacists who completed residency training had a significantly higher adjusted odds of supporting pharmacist-prescribed HC classification vs prescription-only (adjusted odds ratio (aOR) 2.55; 95% CI, 1.18, 5.54; Table 3). Agreeing that pharmacists prescribing HC would strengthen relationships with local physicians was significantly associated with supporting pharmacist-prescribed HC classification vs prescription-only (aOR, 3.37; 95% CI, 1.58, 7.18) and with supporting non-prescription classification vs prescription-only (aOR, 5.50; 95% CI, 2.47, 12.32; Table 3).

**Table 3.** Predictors of Believing Oral Contraception Should be Classified Pharmacist-Prescribed or Non-Prescription vs Prescription-Only.

Predictor Variable	Pharmacist-Prescribed				Non-prescription			
	Bivariate		Multivariate		Bivariate		Multivariate	
	Or (95% CI)	p-value	aOR (95% CI)	p-value	Or (95% CI)	p-value	aOR (95% CI)	p-value
Age < 40 years old (vs 40 years or older)	1.502 (1.005, 2.243)	.047*	1.796 (.668, 4.829)	.246	1.156 (.722, 1.850)	.547	1.291 (.452, 3.686)	.859
Female gender (vs non-female)	1.357 (.893, 2.063)	.152	.814 (.487, 1.361)	.433	.824 (.510, 1.331)	.430	.594 (.342, 1.030)	.075
< 11 years as a pharmacist	1.304 (.877, 1.938)	.189	.524 (.162, 1.693)	.280	1.108 (.695, 1.766)	.666	.704 (.202, 2.454)	.581
11-20 years as a pharmacist	1.120 (.661, 1.898)	.673	.691 (.284, 1.683)	.416	1.070 (.575, 1.989)	.832	.834 (.318, 2.192)	.982
Pharm.D. education	1.590 (1.031, 2.435)	.036*	1.202 (.550, 2.624)	.645	1.155 (.701, 1.901)	.572	1.074 (.464, 2.488)	.9954
Pharmacy residency training	3.314 (1.889, 5.814)	< .001*	2.552 (1.176, 5.538)	.018*	1.596 (.817, 3.119)	.172	1.651 (.684, 3.984)	.275
Further education (fellowship, MS, and PhD)	2.239 (.831, 6.038)	.111	2.220 (.607, 8.120)	.228	1.506 (.467, 4.861)	.493	1.137 (.274, 4.712)	.832
Board certified	2.316 (1.246, 4.305)	.008*	1.016 (.429, 2.409)	.970	1.413 (.674, 2.964)	.360	1.005 (.373, 2.709)	.977
Other education (e.g., certifications)	1.696 (.783, 3.672)	.180	1.482 (.559, 3.930)	.430	1.448 (.590, 3.551)	.419	1.723 (.601, 4.939)	.309
Graduating from a North Carolina school of pharmacy	.815 (.537, 1.237)	.336	.870 (.522, 1.450)	.593	1.309 (.210, 8.165)	.203	.747 (.429, 1.299)	.286
Self-identified as a community pharmacist	.613 (.410, .917)	.017*	1.032 (.554, 1.923)	.921	.716 (.447, 1.147)	.165	.774 (.399, 1.500)	.501
Suburban location of primary pharmacy site (vs urban)	.874 (.537, 1.423)	.589	.795 (.437, 1.444)	.451	1.317 (.734, 2.366)	.356	1.262 (.654, 2.433)	.467
Rural location of primary pharmacy site (vs urban)	.702 (.420, 2.366)	.179	.0.755 (.399, 1.428)	.388	1.022 (.548, 1.907)	.945	1.083 (.534, 2.196)	.806
Sells emergency contraception at their pharmacy	1.065 (1.015 1.118)	.011*	1.015 (.923, 1.116)	.761	1.010 (.954, 1.070)	.722	.943 (.852, 1.043)	.256
Medication therapy management clinical service offered at the pharmacy	.936 (.606, 1.447)	.767	1.413 (.735, 2.715)	.300	1.120 (.677, 1.854)	.659	1.193 (.599, 2.373)	.616
Agreeing that pharmacists are well trained to prescribe HC	5.508 (3.182, 9.535)	< .001*	3.141 (1.674, 5.895)	< .001*	4.464 (2.428, 8.208)	< .001*	2.598 (1.307, 5.166)	.007*
Agreeing prescribing HC allows pharmacists to practice at a higher level	14.702 (7.964, 27.141)	< .001*	8.520 (4.382, 16.566)	< .001*	3.199 (1.879, 5.444)	< .001*	1.857 (1.012, 3.409)	.046*
Agreeing that prescribing HC will strengthen relationships with local physicians and clinics	1.054 (.983, 1.129)	.141	3.369 (1.581, 7.181)	.002*	1.050 (.970, 1.138)	.226	5.501 (2.465, 12.319)	< .001*

Table Notes: Odds compared to prescription-only; bold denotes statistical significance; multivariate model run with all predictor variables in Table 3. Abbreviations—Pharm.D, Doctor of Pharmacy; PGY, post-graduate year; HC, hormonal contraception. *denotes statistically significant p-value at alpha = .05.

## Discussion

Our survey found that North Carolina pharmacists are generally in favor of pharmacist-prescribed hormonal contraceptives, but far fewer prefer classifying hormonal contraceptives as OTC. When adjusting for other variables, pharmacists were more likely to support pharmacist-prescribed HC over prescription-only if they had completed residency training and if they agreed that prescribing HC would allow them to practice at a higher level, improve relationships with local physicians, and that they were well trained to do so. Similarly, pharmacists were only more likely to support non-prescription HC classification over prescription-only if they agreed with the latter 3 sentiments. Pharmacist attitudes toward classification of hormonal contraception did not differ by geographic location.

Most NC pharmacists supported changing the classification of hormonal contraception to pharmacist-prescribed. Allowing pharmacists to prescribe hormonal contraception may remove some barriers that patients currently encounter.^11^ A 2011 nationally representative web-based survey of adult women at risk of unintended pregnancy found that among women who had ever tried to get a prescription for HC, 13% faced challenges getting to an appointment or clinic, 10% did not have a primary care provider or regular clinic, and 12.5% experienced a clinician requiring a clinic visit or preventative care exam before issuing a prescription; however, only 3.5% had difficulty accessing a pharmacy.^11^ Another survey found that 68% of women would utilize pharmacy services to access hormonal contraception (oral, patch, and ring) and/or emergency contraception; among them were low-income and uninsured women, who reported that accessing contraception through the pharmacy would alleviate the costs and operational hours of a clinic that were a barrier to care.^25^

In the immediate 2 years following Oregon's enactment of pharmacist-prescribed hormonal contraception in 2016, pharmacists wrote 10% of all new oral or transdermal birth control prescriptions filled by Oregon Medicaid beneficiaries.^26^ Furthermore, of all the oral or transdermal birth control prescriptions written by pharmacists during that time, nearly 75% were for women who had not used any form of prescription contraception in the month prior, and almost two-thirds had not used any form of prescription contraception in the 3 mo prior.^26^ Notably, Oregon Medicaid reimburses pharmacists for the clinical service of providing HC in addition to reimbursing for the HC itself.

One major barrier that pharmacist-prescribed hormonal contraception encounters is reimbursement. In the survey by Seamon et al, 54% of pharmacists agreed that reimbursement for this service would be a barrier to implementation.^20^ Although pharmacies have established fees associated with the service, only a few states have established insurance reimbursement for patients with Medicaid coverage and even fewer for private insurance.^27^ This may present an obstacle for those who rely on insurance coverage; however, previous research has found that uninsured individuals tend to utilize pharmacist-prescribed avenues for their hormonal contraception over traditional clinical routes.^28^ Continuing to expand insurance reimbursement will serve to facilitate access for more individuals.

Both pharmacist-prescribed and OTC access to HC uphold safe contraception use. ACOG and the World Health Organization (WHO) agree that neither a pelvic exam nor a Pap smear are required for safe prescribing of hormonal contraceptives, and medical organizations agree that most women can self-screen for contraindications to hormonal contraception^29^ (*American Medical Association Resolution D-75.995 (Sub. Res. 507, A-13): Over-the-Counter Access to Oral Contraceptives*).^12,13^ However, removing prescription status from HC could increase the financial burden of its use. Prescribed HC is covered by insurance, and movement away from a prescription may introduce more financial barriers due to limited insurance coverage of non-prescription pharmaceutical products.^18^ Our findings demonstrate that NC pharmacists align with ACOG and APhA in their support for increased access to HC.

The findings in this study indicate that primary support for non-prescription hormonal contraception is less common as compared to pharmacist-prescribed mechanisms. This is consistent with prior work that found most pharmacists were opposed to non-prescription classification of hormonal contraception, and it highlights a divergence between the views of pharmacists and those of major medical associations, which recommend that oral contraceptives be available OTC (*American Medical Association Resolution D-75.995 (Sub. Res. 507, A-13): Over-the-Counter Access to Oral Contraceptives*).^14,12,17^ However, results of this analysis also indicate that the factors associated with pharmacist-prescribed preference were largely the same for OTC classification preference. This suggests that pharmacists who believe that they are well-trained to manage contraception and that pharmacy involvement with contraception management improves relationships with local physicians tend to support greater access to contraception in general. Thus, continuing education aimed at promoting greater implementation of pharmacist-prescribed models may also strengthen beliefs in OTC access.

Studies have shown that women at risk of unintended pregnancy are largely supportive of OTC hormonal contraception and would utilize it if it were available at a reasonable price as there is currently limited insurance coverage of non-prescription products.^30,31,18^ If contraception were classified as OTC, it is possible, although not certain, that many of the pharmacists who support pharmacist-prescribed mechanisms would support this move, at least in part. Investigations are needed into reasons why pharmacists are less supportive of non-prescription HC classification and whether they would ultimately support this practice in their pharmacies.

### Limitations

The results of this study should be interpreted in light of its limitations. Data were collected from a cross-sectional convenience sample of NC pharmacists, which prevent causal explanations and limit the generalizability to other populations. Second, pharmacists could only select one preferred classification for contraception, though they may support more than one classification (e.g., pharmacist-prescribed and OTC access). Additional research is needed to gauge the level of support beyond preferences. Third, pharmacy characteristics are self-reported and may be interpreted differently based on the respondent. Finally, reasons for not supporting other classifications of contraception were not explored in this study; future research is needed to understand these reasons and their implications.

## Conclusions

Our primary finding demonstrates pharmacist support for state-level enactment of pharmacist-prescribed HC in North Carolina. Beliefs that pharmacists are well-trained to prescribe HC, HC prescribing allows pharmacists to practice at a higher level, and that pharmacist-prescribing HC would strengthen relationships with local physicians were associated with increased support for pharmacist-prescribed HC and OTC access. As the laws and regulations around HC continues to evolve locally and nationally, continuing education initiatives for pharmacists promote training and interprofessionalism will serve to improve implementation of improved contraception access at the pharmacy.
